# Retraction: Engineering the optical properties of nickel sulphide thin films by zinc integration for photovoltaic applications

**DOI:** 10.1039/d6ra90076c

**Published:** 2026-07-24

**Authors:** Junaid Younus, Warda Shahzad, Bushra Ismail, Tanzeela Fazal, Mazloom Shah, Shahid Iqbal, Ahmed Hussain Jawhari, Nasser S. Awwad, Hala A. Ibrahium

**Affiliations:** a Department of Chemistry, COMSATS University Islamabad (CUI), Abbottabad Campus 22060 Pakistan bushraismail@cuiatd.edu.pk; b Department of Chemistry, Abbottabad University of Science and Technology (AUST) Abbottabad Pakistan tanzeelafazal@yahoo.com; c Department of Chemistry, Faculty of Science, Grand Asian University Sialkot Pakistan; d Department of Chemistry, School of Natural Sciences (SNS), National University of Science and Technology (NUST) H-12 Islamabad 46000 Pakistan shahidgcs10@yahoo.com; e Department of Chemistry, Faculty of Science, Jazan University P.O. Box 2097 Jazan 45142 Saudi Arabia; f Chemistry Department, Faculty of Science, King Khalid University P.O. Box 9004 Abha 61413 Saudi Arabia; g Biology Department, Faculty of Science, King Khalid University P.O. Box 9004 Abha 61413 Saudi Arabia

## Abstract

Retraction of ‘Engineering the optical properties of nickel sulphide thin films by zinc integration for photovoltaic applications’ by Junaid Younus *et al.*, *RSC Adv.*, 2023, **13**, 27415–27422, https://doi.org/10.1039/D3RA04011A.

The Royal Society of Chemistry hereby wholly retracts this *RSC Advances* article due to concerns with the reliability of the data.

The micrographs in Fig. 3b and d were first published in ref. 1 and described as a different material.

The UV-visible absorbance data in Fig. 4 and 9 have strong similarity. The UV-visible absorbance data in Fig. 8a and 10b have strong similarity.

The authors provided a response and new data but they did not address the concerns and they were not able to provide the original raw data.

Given the significance of these concerns, the findings presented in this paper are no longer reliable.

Shahid Iqbal opposes this retraction decision. The other authors did not respond to any correspondence regarding the retraction of this article. Junaid Younus and Nasser S. Awwad could not be contacted.

This retraction supersedes the information provided in the Expression of concern related to this article.

Signed: Laura Fisher, Executive Editor, *RSC Advances*

Date: 16th July 2026
